# Utilisation of government-subsidised chronic disease management plans and cardiovascular care in Australian general practices

**DOI:** 10.1186/s12875-022-01763-2

**Published:** 2022-06-21

**Authors:** Genevieve Coorey, Anna Campain, John Mulley, Tim Usherwood, Julie Redfern, Mark Harris, Nicholas Zwar, Sharon Parker, Enrico Coiera, David Peiris

**Affiliations:** 1 The George Institute for Global Health, University of New South Wales, Sydney, Australia; 2 School of Health Sciences, Faculty of Medicine and Health, The University of Sydney, Sydney, Australia; 3 Westmead Applied Research Centre, Faculty of Medicine and Health, The University of Sydney, Sydney, Australia; 4 Centre for Primary Health Care and Equity, University of New South Wales, Sydney, Australia; 5 Faculty of Health Sciences and Medicine, Bond University, Gold Coast, Australia; 6 School of Population Health, University of New South Wales, Sydney, Australia; 7 Australian Institute for Health Innovation, Faculty of Medical and Health Sciences, Macquarie University, Sydney, Australia

**Keywords:** Primary health care, Allied health, Chronic conditions, Medication adherence, Cardiovascular disease, Incentive scheme

## Abstract

**Background:**

Government-subsidised general practice management plans (GPMPs) facilitate chronic disease management; however, impact on cardiovascular disease (CVD) is unknown. We aimed to determine utilisation and impact of GPMPs for people with or at elevated risk of CVD.

**Methods:**

Secondary analysis of baseline data from the CONNECT randomised controlled trial linked to Medicare Benefits Schedule (MBS) and Pharmaceutical Benefits Scheme (PBS) claims. Multivariate regression examining the association of GPMP receipt and review with: (1) ≥ 1 MBS-subsidised allied health visit in the previous 24 months; (2) adherence to dual cardioprotective medication (≥ 80% of days covered with a dispensed PBS prescription); and (3) meeting recommended LDL-cholesterol and blood pressure (BP) targets concurrently.

**Results:**

Overall, 905 trial participants from 24 primary health care services consented to data linkage. Participants with a GPMP (46.6%, 422/905) were older (69.4 vs 66.0 years), had lower education (32.3% vs 24.7% high school or lower), lower household income (27.5% vs 17.0% in lowest bracket), and more comorbidities, particularly diabetes (42.2% vs 17.6%) compared to those without a GPMP. After adjustment, a GPMP was strongly associated with allied health visits (odds ratio (OR) 14.80, 95% CI: 9.08–24.11) but not higher medication adherence rates (OR 0.82, 95% CI: 0.52–1.29) nor meeting combined LDL and BP targets (OR 1.31, 95% CI: 0.72–2.38). Minor differences in significant covariates were noted in models using GPMP review versus GPMP initiation.

**Conclusions:**

In people with or at elevated risk of CVD, GPMPs are under-utilised overall. They are targeting high-needs populations and facilitate allied health access, but are not associated with improved CVD risk management, which represents an opportunity for enhancing their value in supporting guideline-recommended care.

**Supplementary information:**

The online version contains supplementary material available at 10.1186/s12875-022-01763-2.

## Introduction

More than 47% of Australians are affected by at least one chronic condition [1], which may require coordination of care across multiple providers. In Australia, chronic conditions are mostly managed in primary health care via a range of providers including general practitioners (GPs), registered nurses, allied health professionals, and Aboriginal Health Practitioners [1]. Australia’s health care system is a hybrid of private and public health care provision [2]. Basic coverage for health care services is provided under Medicare, the universal health care scheme for citizens and other eligible groups [3]. Free or low-cost services are provided by federal, state, and local levels of government; each level has responsibilities that are specific to their jurisdiction and some activities are shared [2]. The Medicare Benefits Schedule (MBS) subsidises most GP consultations and some ambulatory care services including some allied health services; other primary health care tends to be funded by private, state or territory governments, and non-government sources [4]. Health services that are not fully covered under the MBS scheme, or for which the provider charges above the scheduled fee, incur an out-of-pocket (gap) charge [3]; private health insurance may offset some out-of-pocket costs [2]. The federally-funded Pharmaceutical Benefits Scheme (PBS) is a co-payment scheme facilitating prescription medication affordability [3]. The MBS and PBS both have provisions for lowering the out-of-pocket costs for services or medications required by low-income and other eligible groups [3].

For people with chronic health conditions, Medicare subsidises structured assessment, planning, and multidisciplinary care under the chronic disease management plan initiative [5]. Within this initiative, a GP can initiate a general practice management plan (GPMP) which is a documented comprehensive description of the care needs, management goals, action, treatment, and service arrangements and reviews for the relevant condition(s) [6]. Further, a team care arrangement (TCA) documents coordination of care required from at least three providers [6]. The original enhanced primary care initiative for chronic and complex care commenced in 1999 and in 2004–2005 underwent significant expansion of provisions for aged care, Aboriginal health, and allied health access. With a GPMP in place, the recipient is allowed MBS-subsidised attendance of up to five allied health visits per calendar year [7]. Previous research on the impact of GPMP items demonstrated mixed outcomes with limited effectiveness for multi-provider collaboration; they were mainly used for administrative purposes rather than clinical motivations [8]. From 2006–2014 there was an overall doubling in the proportion of initial claims for GPMP or TCA (11.3% to 22.4%), with the highest uptake found: (i) in those aged 80–84 years; (ii) in people with diabetes; (iii) in those with lower physical functioning; and (iv) in people on lower incomes [9].

Formal care planning in chronic conditions has potential to benefit physical and psychological health outcomes, as well as self-care activities [10], and several Australian studies have assessed the impact of GP care plans on hospitalisation outcomes [11–14]. In adults with diabetes, three subsidised items were associated with reduced all-cause hospitalisations: an annual cycle of diabetes care; review but not initiation of a GPMP/TCA; and multidisciplinary care, in particular from ophthalmology, a practice nurse, and podiatry [11]. Confounding factors notwithstanding, active care—expressed as proactive health reviews and early and ongoing preventive care— appeared more protective against hospital admissions than care plan initiation alone. In older adults with heart failure, having a GPMP was associated with a 23% reduction in the rate of preventable heart failure hospitalisation [13]; also delayed time to, and reduced rate of, hospitalisation for any cardiovascular or respiratory cause. In contrast, another study found that for a general population with chronic diseases, there was no association between a GPMP claimed within a two-year baseline service utilisation period and potentially preventable hospitalisations in the subsequent five years [12]. A study looking at uptake of five or more physiotherapy claims under a GPMP in patients aged 85 years or older found this was associated with fewer avoidable hospitalisations [7]. Aside from one heart failure study, the benefit of GPMPs for management of cardiovascular disease (CVD) is uncertain [9, 15]. Given the overall purpose of the GPMP to improve management of chronic care needs, goals, action, treatment, and services, the objectives of this study were to:

1. Identify differences in socio-demographic and clinical characteristics in people with or at elevated risk of CVD by receipt of a GPMP.
2. Determine if there is an association between receipt or review of a GPMP and (i) allied health service utilisation; (ii) adherence to cardiovascular medications; and (iii) attainment of guideline recommended blood pressure (BP) and fasting low-density lipoprotein (LDL) cholesterol targets.

## Methods

Secondary analysis of baseline data from the Consumer Navigation of Electronic Cardiovascular Tools (CONNECT) randomised controlled trial (RCT), which took place in twenty-four primary care services in Sydney, Australia. Study sites were spread across greater Sydney, including the Blue Mountains region, and one service was an Aboriginal Community Controlled Health Service. Practices varied by ownership model, GP numbers, and on-site multidisciplinary services. CONNECT assessed the impact of a consumer web-based application that was linked to a patient’s primary care electronic medical record. Details of the trial and outcomes have previously been reported [16].

### Participants

Eligible RCT participants were defined as any of the following: (1) a five-year CVD risk ≥ 10% using the Framingham risk equation; (2) a clinically high-risk condition (diabetes and age > 60 years, diabetes and albuminuria, estimated glomerular filtration rate < 45 ml/min, systolic BP > 180 mmHg, diastolic BP > 110 mmHg, total cholesterol > 7.5 mmol); or (3) a CVD diagnosis documented in the GP health record. This sub-analysis included only the enrolled participants who provided written informed consent to MBS and PBS data linkage.

### Data sources

Participants were identified by their unique Medicare number for linkage to MBS-funded GP, specialist, and allied health consultations, and receipt of chronic disease management items. (Table 1) Consent for use of their Medicare number for this purpose was provided by participants enrolled in the original clinical trial. Use of the Medicare identifier enabled direct (or deterministic) data linkage relationships between all data sources in the project. Medication dispensing data were derived from the PBS. Medicare and PBS data were obtained from Services Australia at study baseline, with a look-back period of 4.5 years. Demographic and laboratory data, and self-reported health conditions and medication use were obtained from the RCT electronic database.

**Table 1:** MBS-subsidised services and related item numbers^a^

Service description	**Item numbers**
General Practice (GP) consultations	3 23 36 44 4 24 37 47 90001 90020 90035 90043 90051
Out of hours GP consultation	5000 5020 5040 5060 5003 5023 5043 5063 5010 5028 5049 5067 585 599
General Practice Management Plan (GPMP)	721
Team Care Arrangement (TCA)	723
Review of TCA and GPMP	732
Medication management review	900
Service by a practice nurse	10997
Mental health consultation	2713
Mental Health Management Plan	2700 2701 2715 2717 2712
Allied Health	
Diabetes educator	10951
Dietitian	10954
Exercise physiologist	10953
Physiotherapist	10960
Podiatrist	10962
Psychologist	10968 80100 80105 80110 80115 80120
Health Assessment by a GP	701 703 705 707 715 699
Specialist consultation, including psychiatrist	104 105 110 116 119 132 133 291 293

^a^Item numbers for non-vocationally registered GPs comprised < 0.3% of the MBS claims and were omitted from the analyses

### Covariates

Demographic, socioeconomic, lifestyle, clinical characteristics, laboratory data, and self-reported health conditions were obtained from baseline RCT data. Service utilisation variables were constructed from the MBS data and included number of GP appointments, GPMP receipt, GPMP review, specialist services, and allied health services in the 24 months preceding RCT baseline. If two item numbers were claimed for the same appointment, then it was counted as two appointments. Medication adherence was calculated using proportion of days covered (PDC), with adherence requiring PDC ≥ 80%. (see Supplement 1 for technical details on calculation method).

### Outcomes

The pre-specified outcomes of interest were the associations between the provision of a GPMP and:

i. MBS-subsidised allied health services, defined as uptake of at least one service in the previous 24 months
ii. adherence to dual guideline-recommended cardiovascular medications, defined as a PDC of ≥ 80% based on pharmacy dispensing data for both lipid-lowering and antihypertensive medications.
iii. concurrent attainment of Australian guideline-recommended blood pressure and fasting LDL cholesterol targets, defined as ≤ 130/80 mmHg for people with CVD, diabetes or albuminuria and ≤ 140/90 mmHg for all others, and LDL-cholesterol < 2.0 mmol/l.

These associations were also investigated for a GPMP review instead of an initial GPMP receipt. Blood pressure and LDL targets reflect National Vascular Disease Prevention Alliance guidelines used within the CONNECT RCT [17].

### Statistical analyses

Variable selection from the total list of 74 variables was conducted on the basis of a priori clinical relevance and boosted regression tree (BRT) models [18]. A priori variables, selected from review of literature and expert clinician input, included sex, age, education level, income, attainment of clinical targets (when studying medication adherence), medication adherence (when studying attainment of clinical targets), and ten comorbidities (atrial fibrillation, coronary heart disease, chronic kidney disease, chronic obstructive pulmonary disease, heart failure, peripheral vascular disease, stroke, diabetes, depression, and asthma). Three BRT models were developed to quantify the influence of variables used to distinguish the groups with and without a GPMP for the three outcomes of interest. Variables were ranked in order of relative influence and the topmost variables were included in the multivariable regression. Highly correlated variables were removed or interchanged based on the BRT rankings. Multivariable regression was performed using this final subset of variables and practice level random effect. Three additional multivariable models were created using GPMP review instead of GPMP receipt, and retaining the same variables for comparative purposes. Categorical exposure variables were modelled using linear terms and their effects illustrated using forest plots of the odds ratios and 95% confidence intervals displayed with density strips [19].

Variables with missing data were permitted in the BRT models, however, only participants with complete cases for the included variables were used in the multiple regression models. Influential variables with large amounts of missing values were interchanged with more complete variables.

Standard summary statistics were used throughout, including measures of proportions, measures of central tendency (median and mean) and dispersion (the interquartile range and standard deviation). The analysis was performed using both SAS version 9.4, SAS Enterprise Guide 7.1 and R version 3.6.2 [20] including R packages gbm 2.1.5, mgcv 1.8–28.

### Ethics

Approval for the CONNECT RCT was from the Human Research Ethics Committees of the University of Sydney (Reference 2013/716); the New South Wales Aboriginal Health and Medical Research Council (Reference 959/13); and the Australian Department of Health (Reference 16/2014).

## Results

Linked data were available for 905 participants (96.9% of the overall RCT cohort). At baseline, 46.6% of participants (422/905) had a GPMP recorded. Of those participants, 39.3% (166/422) had a GPMP review recorded. Overall, GPMPs were directed toward high needs participants including people who were older (69.4 vs 66.0 years), had lower education (32.3% vs 24.7% high school or lower), lower household income (27.5% vs 17.0% in lowest bracket), and more comorbidities, particularly diabetes (42.2% vs 17.6%) compared to those without a GPMP. (Tables 1 and 2) In unadjusted comparisons to those without a GPMP, participants with a GPMP had lower mean total cholesterol, lower mean LDL cholesterol, lower mean diastolic BP and systolic BP, and higher mean fasting blood glucose. (Table 3) Further, more people with a GPMP than without one had LDL cholesterol controlled (35.1% vs 21.5%) and had both BP and LDL cholesterol controlled (14.9% vs 7.5%). (Table 4) Adherence to lipid-lowering and anti-hypertensive medications (self-reported and assessed by PDC) was higher in those with a GPMP. (Table 4).

**Table 2:** Sociodemographic characteristics of participants with and without a GPMP in the 24 months preceding RCT enrolment

Characteristic	With a GPMP (*N* = 422)	Without a GPMP (*N* = 483)	Total (*N* = 905)
Age (yrs): Mean (SD)	69.4 (7.6)	66.0 (8.0)	67.6 (8.0)
Male	293/422 (69.4%)	400/483 (82.8%)	693/905 (76.6%)
Ethnicity			
Caucasian	352/422 (83.4%)	431/483 (89.2%)	783/905 (86.5%)
Aboriginal or Torres Strait Islander	27/422 (6.4%)	8/483 (1.7%)	35/905 (3.9%)
Asian	21/422 (5.0%)	12/483 (2.5%)	33/905 (3.6%)
Other	22/422 (5.2%)	32/483 (6.6%)	54/905 (6.0%)
Education level			
None	1/421 (0.2%)	0/482(0.0%)	1/903 (0.1%)
Primary school	17/421 (4.0%)	10/482 (2.1%)	27/903 (3.0%)
Secondary school	118/421 (28.0%)	109/482 (22.6%)	227/903 (25.1%)
Undergraduate degree	81/421 (19.2%)	98/482 (20.3%)	179/903 (19.8%)
Postgraduate degree or diploma	99/421 (23.5%)	148/482 (30.7%)	247/903 (27.4%)
Technical/vocational qualification	105/421 (24.9%)	117/482 (24.3%)	222/903 (24.6%)
Household income			
$0–799/week	116/422 (27.5%)	82/481 (17.0%)	198/903 (21.9%)
$800–1999/week	151/422 (35.8%)	176/481 (36.6%)	327/903 (36.2%)
> $2000/week	78/422 (18.5%)	149/481 (31.0%)	227/903 (25.1%)
No response	77/422 (18.2%)	74/481 (15.4%)	151/903 (16.7%)
SEIFA			
LEVEL 1—2	13/421 (3.1%)	12/481 (2.5%)	25/902 (2.8%)
LEVEL 3—4	24/421 (5.7%)	12/481 (2.5%)	36/902 (4.0%)
LEVEL 5—6	27/421 (6.4%)	29/481 (6.0%)	56/902 (6.2%)
LEVEL 7—8	85/421 (20.2%)	102/481 (21.2%)	187/902 (20.7%)
LEVEL 9—10	272/421 (64.6%)	326/481 (67.8%)	598/902 (66.3%)
Insurance Status			
Private Health Insurance	320/421 (76.0%)	407/483 (84.3%)	727/904 (80.4%)
Health care card or DVA card	230/421 (54.6%)	183/482 (38.0%)	413/903 (45.7%)
Employment			
Full-time	62/422 (14.7%)	149/479 (31.1%)	211/901 (23.4%)
Part-time	57/422 (13.5%)	55/479 (11.5%)	112/901 (12.4%)
Retired	281/422 (66.6%)	262/479 (54.7%)	543/901 (60.3%)
Not working	22/422 (5.2%)	13/479 (2.7%)	35/901 (3.9%)
Relationship status			
Married/defacto	308/422 (73.0%)	400/482 (83.0%)	708/904 (78.3%)
Single	30/422 (7.1%)	30/482 (6.2%)	60/904 (6.6%)
Divorced	44/422 (10.4%)	25/482 (5.2%)	69/904 (7.6%)
Widowed	40/422 (9.5%)	27/482 (5.6%)	67/904 (7.4%)

*Abbreviations*: *DVA* Department of Veterans' Affairs, *GPMP* General practice management plan, *SEIFA* Socio-Economic Indexes for Areas

**Table 3:** Clinical and related characteristics of participants with and without a GPMP in the 24 months preceding RCT enrolment

	**With a GPMP (*N* = 422)**	**Without a GPMP (*N* = 483)**	**Total (*N* = 905)**
**Biometric**			
BMI (kg/m2) (mean (SD))	29.9 (5.5)	29.7 (5.2)	29.8 (5.3)
Waist circumference (cm) (mean (SD))	106.4 (14.4)	105.7 (14.2)	106.0 (14.3)
Systolic BP (mmHg) (mean (SD))	137.0 (16.7)	139.1 (15.9)	138.2 (16.3)
Diastolic BP (mmHg) (mean (SD))	77.6 (10.8)	80.8 (10.3)	79.3 (10.6)
**Clinical**			
Fasting glucose (mmol/L) (mean (SD))	6.3 (1.9)	5.8 (1.7)	6.1 (1.8)
Urinary albumin to creatinine ratio (mg/mmol) (median, (IQR))	4.0 (17.8)	1.9 (3.8)	2.9 (12.6)
Serum creatinine umol/L (mean (SD))	85.2 (36.8)	85.9 (45.0)	85.6 (41.3)
Total cholesterol (mean (SD))	4.5 (1.1)	4.8 (1.2)	4.6 (1.1)
LDL (mean (SD))	2.4 (0.9)	2.8 (1.0)	2.6 (1.0)
HDL (mean (SD))	1.3 (0.4)	1.3 (0.4)	1.3 (0.4)
Triglycerides (mean (SD))	1.5 (0.9)	1.6 (1.1)	1.6 (1.0)
HbA1C (mean (SD))	6.8 (1.3)	6.9 (1.3)	6.8 (1.3)
Number of daily medications (median (IQR))	5.0 (3.0, 8.0)	3.0 (2.0, 6.0)	4.0 (2.0, 7.0)
**Comorbidities**			
Previous stroke	49/422 (11.6%)	37/483 (7.7%)	86/905 (9.5%)
Coronary heart disease	161/421 (38.2%)	141/483 (29.2%)	302/904 (33.4%)
Peripheral vascular disease	20/421 (4.8%)	13/483 (2.7%)	33/904 (3.7%)
Atrial fibrillation	62/421 (14.7%)	37/483 (7.7%)	99/904 (11.0%)
Heart failure	8/422 (1.9%)	2/483 (0.4%)	10/905 (1.1%)
Diabetes	178/422 (42.2%)	85/483 (17.6%)	263/905 (29.1%)
COPD/emphysema	34/421 (8.1%)	24/483 (5.0%)	58/904 (6.4%)
Chronic kidney disease	13/422 (3.1%)	14/483 (2.9%)	27/905 (3.0%)
Asthma	61/421 (14.5%)	62/482 (12.9%)	123/903 (13.6%)
Depression/Anxiety	87/421 (20.7%)	91/483 (18.8%)	178/904 (19.7%)
**Diet and Lifestyle**			
Servings of fruit per week (mean (SD))	11.4 (8.5)	11.2 (8.5)	11.3 (8.5)
Servings of vegetables per week (mean (SD))	16.2 (9.7)	15.7 (9.6)	15.9 (9.6)
Servings of fish per week (mean (SD))	1.4 (1.3)	1.3 (1.1)	1.4 (1.2)
Use of olive oil for cooking	307/418 (73.4%)	331/479 (69.1%)	638/897 (71.1%)
Sodium control in diet	309/420 (73.6%)	344/477 (72.1%)	653/897 (72.8%)
Current smoker (self-reported or CO reading > 8 ppm)	50/417 (12.0%)	65/476 (13.7%)	115/893 (12.9%)
Alcohol consumption per week (standard drinks): Median (Q1, Q3)	2.0 (0.0, 10.0)	5.0 (0.0, 14.0)	4.0 (0.0, 12.0)
Quality of life (mean EQ5D visual analogue score (SD))	79.0 (15.0)	80.8 (13.6)	80.0 (14.3)
GPAQ Sedentary metric: Mean (SD)	340.4 (184.1)	366.2 (186.3)	354.2 (185.6)
**Health Literacy Scales (selected measures)**			
Feeling understood and supported by healthcare providers: Mean (SD)	3.4 (0.4)	3.3 (0.5)	3.3 (0.4)
Having sufficient information to manage my health: Mean (SD)	2.9 (0.5)	2.9 (0.4)	2.9 (0.5)
Actively managing my health: Mean (SD)	2.9 (0.4)	2.9 (0.5)	2.9 (0.5)
Social support for health: Mean (SD)	3.1 (0.5)	3.1 (0.4)	3.1 (0.5)
Appraisal of health information: Mean (SD)	2.8 (0.5)	2.8 (0.5)	2.8 (0.5)
Ability to actively engage with healthcare providers: Mean (SD)	4.3 (0.5)	4.3 (0.5)	4.3 (0.5)
Navigating the healthcare system: Mean (SD)	4.1 (0.5)	4.1 (0.5)	4.1 (0.5)
Ability to find good health information: Mean (SD)	4.0 (0.6)	4.0 (0.5)	4.0 (0.5)
Understand health information well enough to know what to do: Mean (SD)	4.2 (0.5)	4.2 (0.5)	4.2 (0.5)

*Abbreviations: BMI* Body mass index, *BP* Blood pressure, *CO* Carbon monoxide, *COPD*, Chronic obstructive pulmonary disease, *GPAQ* Global Physical Activity Questionnaire, *GPMP* General practice management plan, *HbA1c* Glycosylated haemoglobin, *HDL* High density lipoprotein, *IQR* Interquartile range, *LDL* Low density lipoprotein

**Table 4:** Service utilisation and CVD management for participants with and without a GPMP in the 24 months preceding RCT enrolment

	**With a GPMP** (***N* = 422)**	**Without a GPMP** (***N* = 483)**	**Total** (***N* = 905)**
**Service Provision**			
Number of GP appointments (median (IQR))	19.0 (12.0, 27.0)	12.0 (8.0, 17.0)	14.0 (10.0, 22.0)
Seen by a practice nurse	112/422 (26.5%)	14/483 (2.9%)	126/905 (13.9%)
At least one specialist consultation	389/422 (92.2%)	399/483 (82.6%)	788/905 (87.1%)
**MBS Items Claimed**			
Health assessment	81/422 (19.2%)	23/483 (4.8%)	104/905 (11.5%)
Team Care Arrangement	380/422 (90.0%)	3/483 (0.6%)	383/905 (42.3%)
Mental Health Management Plan	35/422 (8.3%)	26/483 (5.4%)	61/905 (6.7%)
Review of GPMP/TCA	166/422 (39.3%)	27/483 (5.6%)	193/905 (21.3%)
Mental Health Consultation	35/422 (8.3%)	13/483 (2.7%)	48/905 (5.3%)
Medication review	11/422 (2.6%)	6/483 (1.2%)	17/905 (1.9%)
**MBS-subsidised Allied Health Professional Visits (at least once)**			
Diabetes educator	1/422 (0.2%)	0/483(0.0%)	1/905 (0.1%)
Dietitian	49/422 (11.6%)	13/483 (2.7%)	62/905 (6.9%)
Exercise physiologist	29/422 (6.9%)	2/483 (0.4%)	31/905 (3.4%)
Podiatrist	158/422 (37.4%)	15/483 (3.1%)	173/905 (19.1%)
Physiotherapist	111/422 (26.3%)	19/483 (3.9%)	130/905 (14.4%)
Psychologist	17/422 (4.0%)	17/483 (3.5%)	34/905 (3.8%)
Any of the above	293/422 (69.4%)	56/483 (11.6%)	349/905 (38.6%)
**Guideline Recommended Medication Use (self-report or PDC last 12 months)**			
BP-lowering (self-report)^a^	268/422 (63.5%)	248/483 (51.3%)	516/905 (57.0%)
BP-lowering (prescription data)^b^	251/422 (59.5%)	221/483 (45.8%)	472/905 (52.2%)
Lipid-lowering (self-report)^a^	240/422 (56.9%)	225/483 (46.6%)	465/905 (51.4%)
Lipid-lowering (prescription data)^b^	190/422 (45.0%)	150/483 (31.1%)	340/905 (37.6%)
Combined BP and lipid-lowering (prescription data)	149/422 (35.3%)	106/483 (21.9%)	255/905 (28.2%)
**Meeting Clinical Targets**			
BP^c^	169/422 (40.0%)	179/483 (37.1%)	348/905 (38.5%)
LDL^d^	148/422 (35.1%)	104/483 (21.5%)	252/905 (27.8%)
Both BP and LDL targets met	63/422 (14.9%)	36/483 (7.5%)	99/905 (10.9%)

^a^Defined as taking the medication for five or more of the previous seven days

^b^Defined as PDC of ≥ 80% based on pharmacy dispensing data

^c^ Defined as ≤ 130/80 mmHg for people with CVD, diabetes or albuminuria and ≤ 140/90 mmHg for all others

^d ^LDL-cholesterol < 2.0 mmol/l

*Abbreviations*: *BP* Blood pressure, *GP* General practice, *GPMP* General practice management plan, *IQR* Interquartile range, *LDL* Low density lipoprotein, *PBS* Pharmaceutical Benefits Scheme, *PDC* Proportion of days covered

### Engagement with allied health services

**Fig. 1:**
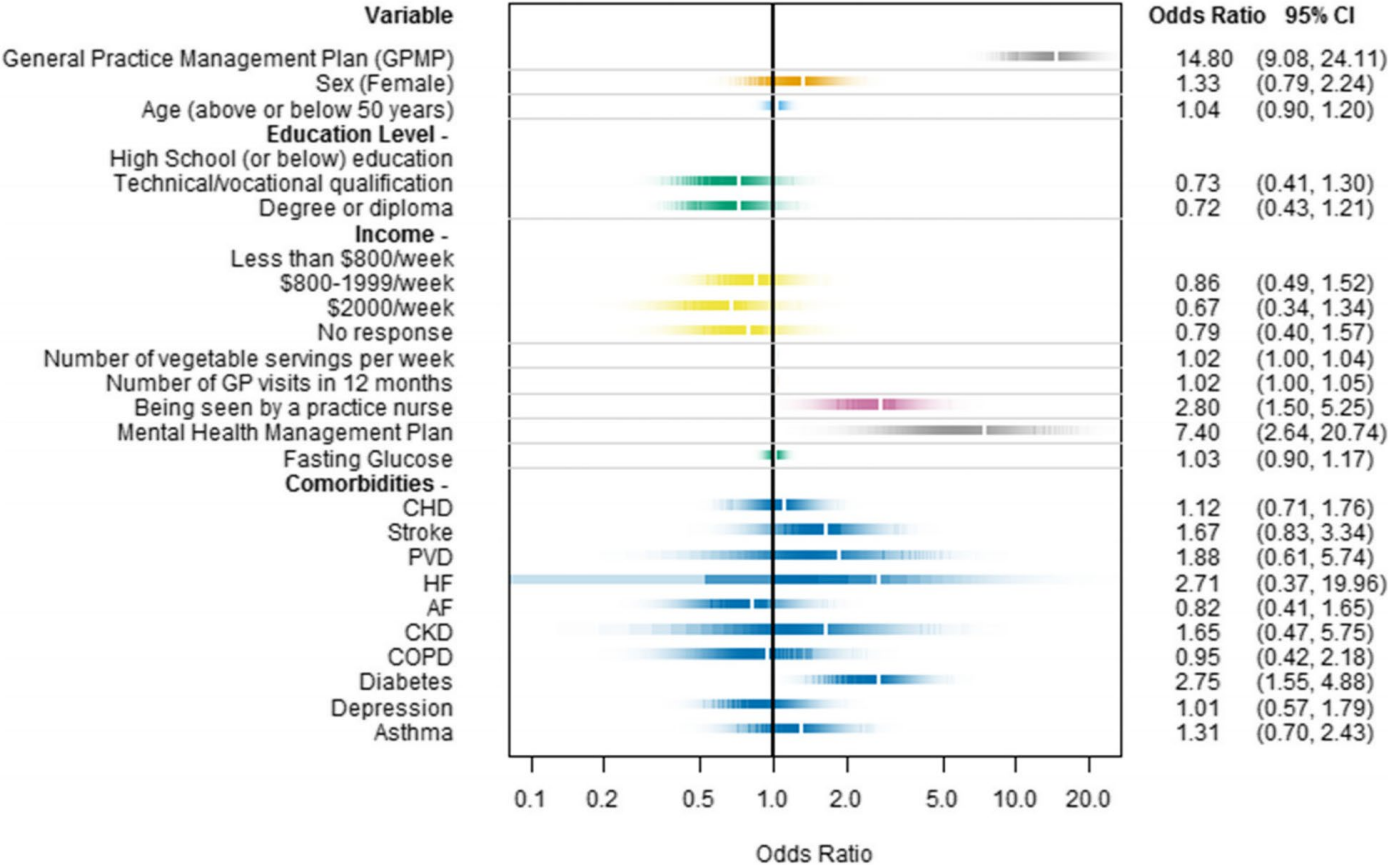
Multivariable logistic regression for use of allied health services with a practice level random effect and adjusted odds ratios. Abbreviations: AF, atrial fibrillation; CHD, coronary heart disease; CKD, chronic kidney disease; COPD, chronic obstructive pulmonary disease; GP, general practice; HF, heart failure; PVD, peripheral vascular disease

Additional BRT ranked variables for use of allied health services in the 24 months prior to RCT enrolment included: number of vegetable servings per week, number of GP visits, GPMP review, being seen by a practice nurse, having a mental health management plan (MHMP), and fasting blood glucose. (Supplement 2) These variables, and those selected a priori (sex, age, education level, income, and 10 comorbidities), were included in the regression model. After adjusting for these co-variates, a GPMP was strongly associated with allied service use (OR 14.80, 95% CI: 9.08, 24.11). Other notable factors associated with allied health service use included receipt of a MHMP (OR 7.40, 95% CI: 2.64, 20.74); being seen by a practice nurse (OR 2.80, 95% CI: 1.50, 5.25); and having diabetes (OR 2.75, 95% CI: 1.55, 4.88) (Fig. 1). There was no evidence of an interaction effect between number of GP visits and GPMP receipt. When GPMP review was used instead of GPMP receipt, the three factors remained important in addition to the presence of peripheral vascular disease. (Supplement 3).

### Adherence to dual cardiovascular medications

**Fig. 2:**
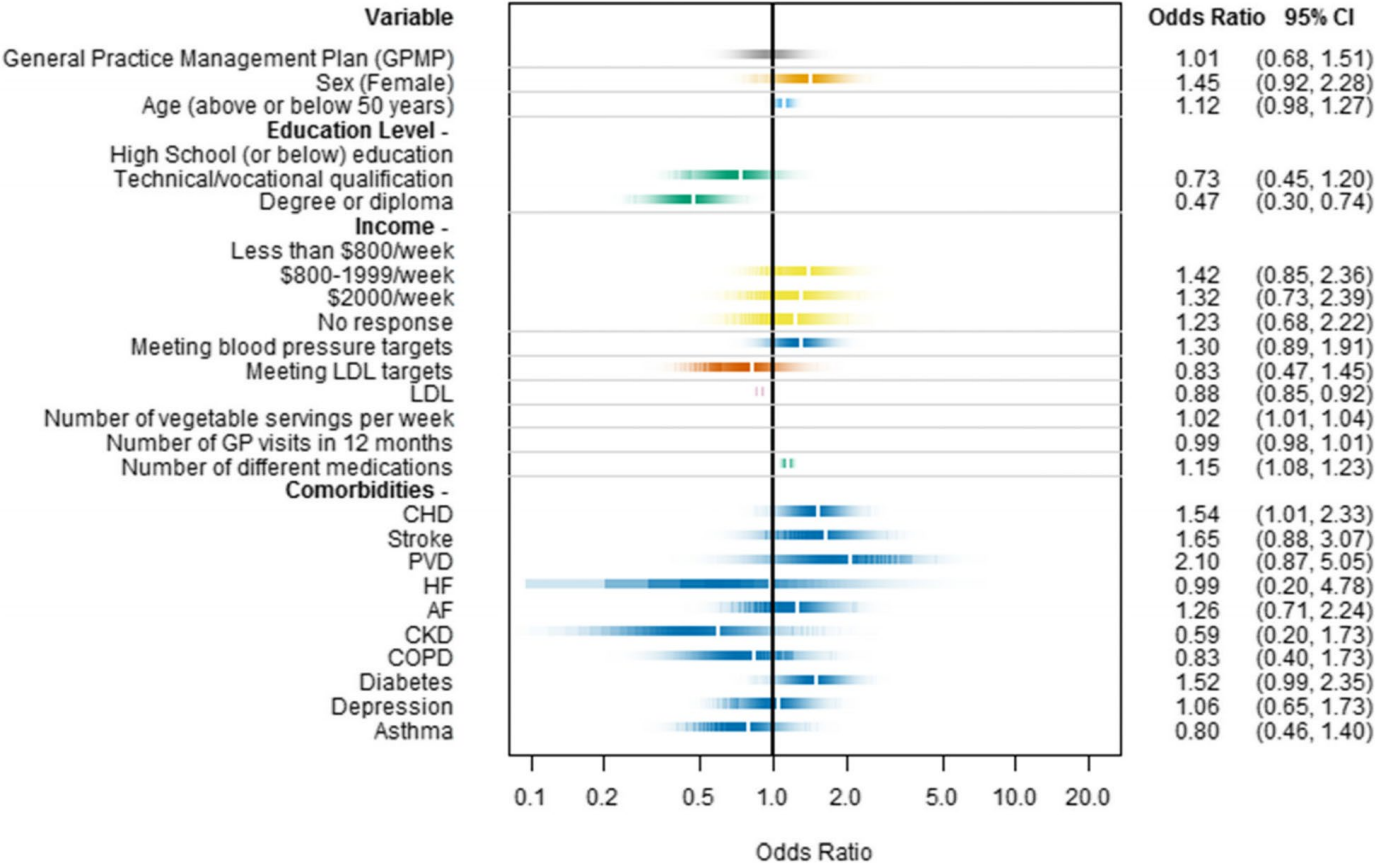
Multivariable logistic regression for adherence to both lipid-lowering and antihypertensive medications with a practice level random effect and adjusted odds ratios. Abbreviations: AF, atrial fibrillation; CHD, coronary heart disease; CKD, chronic kidney disease; COPD, chronic obstructive pulmonary disease; GP, general practice; HF, heart failure; LDL, low density lipoprotein; PVD, peripheral vascular disease

BRT ranked variables for medication adherence in the past 12 months included a person’s LDL cholesterol, number of vegetable servings per week, number of GP visits in the past 24 months, and the number of self-reported medications. (Supplement 2) After adjusting for these variables and those selected a priori (sex, age, education level, income, and 10 comorbidities), a GPMP was not associated with improved medication adherence (OR 0.82, 95% CI: 0.52, 1.29). Other covariates associated with improved adherence included total number of (self-reported) medicines (OR 1.20, 95% CI: 1.11, 1.29), a diagnosis of coronary heart disease (CHD) (OR 1.52, 95% CI: 0.98, 2.35), and increased vegetable consumption (OR 1.02, 95% CI: 1.00, 1.04). Having degree- or diploma-level education was associated with lower adherence compared to having high school or lower education (OR 0.47, 95% CI: 0.29, 0.75). (Fig. 2) There was no evidence of an interaction effect between the number of GP visits and GPMP receipt. When GPMP review was used instead of GPMP receipt, these same four covariates had similar association and the presence of diabetes was a further notable factor. (Supplement 3).

### Attainment of blood pressure and LDL cholesterol targets concurrently

**Fig. 3:**
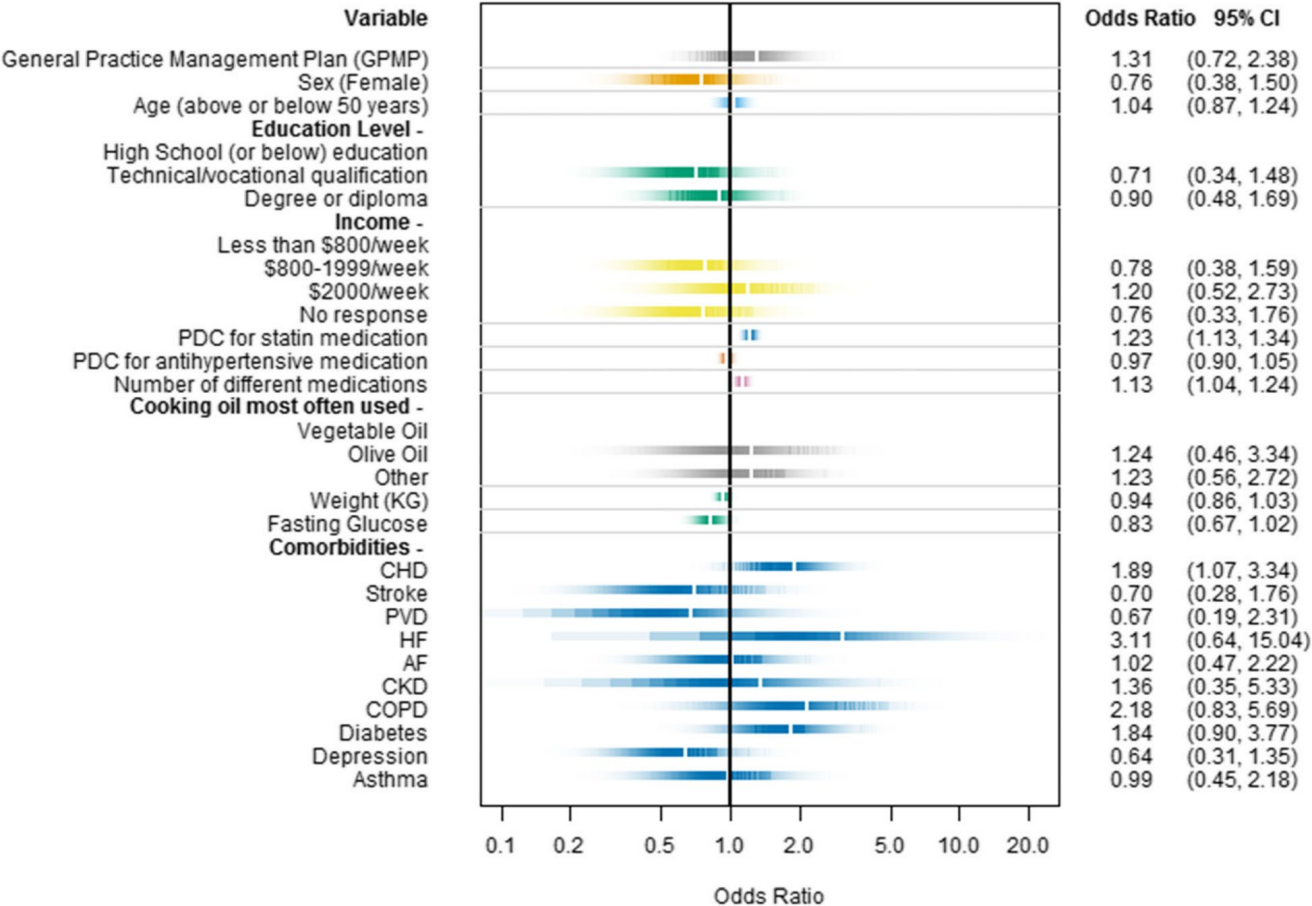
Multivariable logistic regression for attaining both blood pressure and LDL cholesterol targets with a practice level random effect and adjusted odds ratios. Abbreviations: AF, atrial fibrillation; CHD, coronary heart disease; CKD, chronic kidney disease; COPD, chronic obstructive pulmonary disease; HF, heart failure; PDC, proportion of days covered; PVD, peripheral vascular disease

BRT ranked variables for attainment of targets included the number of self-reported medications, the cooking oil most commonly used, weight, and fasting blood glucose. (Supplement 2) After adjusting for these variables and those selected a priori (sex, age, education level, income, and 10 comorbidities)*,* a GPMP was not associated with higher attainment of combined BP and LDL cholesterol targets (OR 1.31, 95% CI: 0.72, 2.38). Other covariates associated with attainment of targets included a diagnosis of CHD (OR 1.89, 95% CI: 1.07, 3.34), the number of medications taken (OR 1.13, 95% CI: 1.04, 1.24), and adherence to statin medication (OR 1.23, 95% CI: 1.13, 1.34). (Fig. 3) When GPMP review was used instead of GPMP receipt, these three factors remained important, and the presence of diabetes was a further notable factor. (Supplement 3).

## Discussion

This study examined baseline data from the CONNECT RCT to evaluate the relationship between provision of a GPMP and uptake of multidisciplinary care, adherence to medications, and attainment of clinical targets in patients with or at elevated risk of CVD. Overall, nearly half (46.6%) of participants had a GPMP claimed in the previous 24 months. People with a GPMP tended to be older, more financially disadvantaged, and have more comorbidities compared to those without a GPMP. This suggests GPs are targeting this service to people who might benefit most, although arguably the whole study population at high risk of CVD could be considered high need. Those with a GPMP had higher GP visit frequency, increased use of health assessments, mental health care plans, and nearly six-fold higher uptake of allied health services. However, neither GPMP receipt nor review, nor visit frequency was associated with medication adherence and clinical targets. We contend that the plan could be strengthened as a tool for greater risk factor control in the context of chronic disease management. Interestingly, although 42.2% of GPMP recipients had diabetes, less than 12% of all allied health claims were for diabetes education or dietitian services. Most claims (63.7%) were for podiatry and physiotherapy, perhaps reflecting personal preferences and/or the use of other channels to obtain services. These findings build on a previous study using GP electronic medical record data where we lacked access to patient socio-demographic data and linkage to PBS records [21].

Conversely, a GPMP was not associated with improved adherence to guideline recommended medications or attainment of clinical targets. Although GPMPs are intended to encompass care assessment, agreed management goals, actions, and treatment needs [6], they appear mainly to be used as a pathway to MBS-subsidised multidisciplinary care. To achieve their broader intent, GPMPs may need a more explicit focus on supporting guideline-recommended care, with an emphasis on quality use of medications and lifestyle recommendations that are known to substantially reduce CVD risk. Within the recently launched primary health care 10-year plan, Voluntary Patient Registration with a general practice is an included reform to strengthen continuity of care and improve health outcomes. This will include payment reforms to support preventive care, quality improvement, and quarantining payment for chronic disease management to the registering practice [22]. This provides opportunities to (re-)emphasise disease management within GPMPs, in line with their stated purpose.

In contrast with previous studies, the number of GP visits did not influence adherence to lipid-lowering medications [23, 24]. A more granular evaluation of factors beyond our study scope including recency of diagnoses, visit regularity (in addition to visit frequency) and care continuity could help understand this complex association. Neither GPMP initiation nor review was associated with higher rates of dispensed CVD medication. This is consistent with published literature suggesting that multifaceted provider and consumer focussed strategies are needed to address this challenge [15]. The number of medication reviews claimed in this study was minor and their role in the CVD outcomes of interest could not be determined. Although physician time spent discussing medications with patients may signal improved processes of care, the benefit may apply to particular subgroups such as older age and type of CVD [25]. Innovative tools to overcome adherence barriers include patient portals [16], smartphone applications that facilitate self-monitoring, alerts/reminders, and enhanced interaction with health care professionals [26]. Although not currently incorporated into a GPMP, such activities offer possibilities for modifying the approach to CVD medication management.

There was also no association between a GPMP, or a GPMP review, and attainment of both BP and lipid control targets. Similarly, an Australian trial of an individualised care plan, implemented by the GP and a nurse, and formatted and reimbursed similarly to the existing GP care plan scheme for chronic conditions, did not achieve attainment of six national cardiometabolic risk factor targets in secondary stroke prevention [27]. In the cohort with elevated CVD risk within this analysis, the number of different medications required and prescription-filling behaviour were associated with attainment of risk factor control. This again underscores the importance of proactive medication adherence management plans to minimise barriers to optimal use of medicines. Improved control of LDL cholesterol and BP has been reported with, for example, mobile phone-based interventions [28]. When teamed with other government-supported programs such as home medicines reviews, practice quality improvement incentive programs, and primary health network quality improvement collaboratives, GPMPs could be optimised for medication adherence. Within multifaceted approaches, substantial and cost-effective reductions in CVD burden are possible [29].

Study limitations include that the participants in this study who received a GPMP for a non-CVD condition – for example participants at high risk for CVD but with another diagnosed chronic condition—may have attained important outcomes that are unknown from these data. However, it is important to note in Table 1that the vast majority of people both with and without CVD had a large range of comorbid chronic conditions and that several of the clinical criteria for classification of high-risk CVD include type 2 diabetes, chronic kidney disease, and presence of albuminuria. Also, allied health services delivered privately or within public hospitals such as outpatient cardiac rehabilitation, may not attract MBS reimbursement and will lead to an underestimate of the overall use of allied health care in this study. Oral antiplatelet medications for CVD prevention were omitted from the analyses because the widely used first-line treatment (aspirin) can be obtained without a prescription, unlike agents added for treatment intensification. The PBS does not collect data about over-the-counter medications, such as aspirin, or about medications purchased via private prescriptions. Notably, more than half (54.6%) of the participants in this study with a GPMP were health care- or DVA card holders; one advantage of obtaining medications via the PBS is the contribution these make toward eligibility for the Safety Net entitlement – both for concession card holders and general patients [30]. A consumer may prefer a private prescription, perhaps to avoid a prescription record in the PBS; however, private prescriptions for lipid- and blood pressure-lowering medications were unlikely to be influential in this study context and were not explored. The CONNECT trial took place in primary care services within Sydney, and use of enhanced primary care services has been noted to be lower in metropolitan than regional areas [5].

## Conclusions

In conclusion, although under-utilised overall, government-subsidised GPMPs initiated for a chronic condition in a population at high CVD risk were prioritised for the highest needs patients and primarily to support access to multidisciplinary care. They were not associated with improvements in guideline-recommended CVD medication use and risk factor control. As the government launches its primary health care 10-year plan, an increased emphasis is recommended on using GPMPs to proactively improve guideline-recommended care for people with diagnosed CVD, or with one or more other chronic conditions and at elevated risk of CVD. GPMPs were designed to improve provision of chronic disease care needs, management goals, action, treatment, and service arrangements and reviews; yet this study found little evidence that they are impacting these broader elements and have become mainly a conduit to accessing allied health services. We contend that a more comprehensive application of the GPMP such as shared decision-making, comprehensive chronic care management, and team-based support could increase their value beyond allied health use.

## Supplementary information

**Additional file 1.**

## Data Availability

The datasets generated and/or analysed during the current study are not publicly available due to confidentiality of RCT participants but are available from the corresponding author on reasonable request.

## Abbreviations

BP: Blood pressure
BRT: Boosted regression tree
CHD: Coronary heart disease
CVD: Cardiovascular disease
GP: General practitioner
GPMP: General practice management plan
LDL: Low-density lipoprotein
MBS: Medicare Benefits Schedule
PBS: Pharmaceutical Benefits Scheme
PDC: Proportion of days covered
RCT: Randomised controlled trial
TCA: Team care arrangement(s)

## References

[1] Australian Institute of Health and Welfare. AIHW. 2020. Chronic conditions and multimorbidity. https://www.aihw.gov.au/reports/australias-health/chronic-conditions-and-multimorbidity. Accessed 26 March 2021.

[2] Australian Government Department of Health. 2019. The Australian health system. https://www.health.gov.au/about-us/the-australian-health-system. Accessed 24 Feb 2022.

[3] Willis E, Willis E, Reynolds L, Keleher H (2009). The Australian health care system. Understanding the Australian health care system.

[4] Australian Institute of Health and Welfare. AIHW. 2020. Primary health care. https://www.aihw.gov.au/reports/australias-health/primary-health-care. Accessed 23 March 2021.

[5] Australian Institute of Health and Welfare. AIHW; 2020 Medicare-subsidised GP, allied health and specialist health care across local areas: 2013–14 to 2018–19. https://www.aihw.gov.au/reports/primary-health-care/medicare-subsidised-health-local-areas-2019. Accessed 23 March 2021.

[6] Commonwealth of Australia. 2021 Medicare benefits schedule. http://www9.health.gov.au/mbs/fullDisplay.cfm?type=note&q=AN.0.47&qt=noteID. Accessed 4 Dec 2021.

[7] Barr ML, Welberry H, Comino EJ (2019). Understanding the use and impact of allied health services for people with chronic health conditions in Central and Eastern Sydney, Australia: a five-year longitudinal analysis. Prim Health Care Res Dev.

[8] Vitry AI, Roughead EE, Ramsay EN (2012). Chronic disease management: does the disease affect likelihood of care planning?. Aust Health Rev.

[9] Welberry H, Barr ML, Comino EJ (2019). Increasing use of general practice management and team care arrangements over time in New South Wales. Australia Aust J Prim Health.

[10] Coulter A, Entwistle VA, Eccles A, Ryan S, Shepperd S, Perera R. Personalised care planning for adults with chronic or long-term health conditions. Cochrane Database Syst Rev. 2015;2015(3):CD010523. 10.1002/14651858.CD010523.pub2 PMC6486144 25733495

[11] Comino EJ, Islam MF, Tran DT (2015). Association of processes of primary care and hospitalisation for people with diabetes: a record linkage study. Diabetes Res Clin Pract.

[12] Welberry H, Barr ML, Comino EJ (2019). Do general practice management and/or team care arrangements reduce avoidable hospitalisations in Central and Eastern Sydney, Australia?. BMC Health Serv Res.

[13] Vitry AI, Nguyen TA, Ramsay EN (2014). General practitioner management plans delaying time to next potentially preventable hospitalisation for patients with heart failure. Intern Med J.

[14] Caughey GE, Vitry AI, Ramsay EN (2016). Effect of a general practitioner management plan on health outcomes and hospitalisations in older patients with diabetes. Inter Med J.

[15] Smith SM, Wallace E, O'Dowd T (2021). Interventions for improving outcomes in patients with multimorbidity in primary care and community settings. Cochrane Database Syst Rev.

[16] Redfern J, Coorey G, Mulley J (2020). A digital health intervention for cardiovascular disease management in primary care: CONNECT randomized controlled trial. NPJ Digit Med.

[17] National Vascular Disease Prevention Alliance; 2012 Guidelines for the management of Absolute cardiovascular disease risk. https://www.heartfoundation.org.au/getmedia/4342a70f-4487-496e-bbb0-dae33a47fcb2/Absolute-CVD-Risk-Full-Guidelines_2.pdf. Accessed 27 May 2022.

[18] Elith J, Leathwick JR, Hastie T (2008). A working guide to boosted regression trees. J Anim Ecol.

[19] Jackson CH (2008). Displaying uncertainty with shading. Am Stat.

[20] Team RC (2014). R: a language and environment for statistical computing.

[21] Redfern J, Hyun K, Atkins E (2017). Utilisation of Medicare-funded schemes for people with cardiovascular disease. Aust J Prim Health.

[22] Medicare Benefits Schedule Review Taskforce. Australian Government; 2018 General practice and primary care clinical committee: phase 2. 2018. https://www1.health.gov.au/internet/main/publishing.nsf/Content/mbs-review-2018-taskforce-reports-cp/%24File/General-Practice-and-Primary-Care-Clinical-Committee-Phase-2-Report.pdf. Accessed 10 Dec 2021.

[23] Ahmed ST, Mahtta D, Rehman H (2020). Association between frequency of primary care provider visits and evidence-based statin prescribing and statin adherence: findings from the Veterans Affairs system. Am Heart J.

[24] Dobbins JM, Elliott SW, Cordier T (2019). Primary care provider encounter cadence and HbA1c control in older patients with diabetes. Am J Prev Med.

[25] Kong E, Beshears J, Laibson D (2020). Do physician incentives increase patient medication adherence?. Health Serv Res.

[26] Agher D, Sedki K, Tsopra R (2020). Influence of connected health interventions for adherence to cardiovascular disease prevention: a scoping review. Appl Clin Inf.

[27] Olaiya MT, Cadilhac DA, Kim J (2017). Community-based intervention to improve cardiometabolic targets in patients with stroke. Stroke.

[28] Palmer MJ, Machiyama K, Woodd S (2021). Mobile phone-based interventions for improving adherence to medication prescribed for the primary prevention of cardiovascular disease in adults. Cochrane Database Syst Rev.

[29] Patel B, Peiris DP, Patel A (2020). A computer-guided quality improvement tool for primary health care: cost-effectiveness analysis based on TORPEDO trial data. Med J Aust.

[30] Australian Government Department of Health. 2022. PBS frequently asked questions. https://www.pbs.gov.au/info/general/faq#HowmuchamIchargedfornonPBSitems. Accessed 01 April 2022.

