# Supplementary material for: Utilisation of government-subsidised chronic disease management plans and cardiovascular care in Australian general practices
Source: BMC Prim Care. 2022 Jun 21;23:157. doi: 10.1186/s12875-022-01763-2 (PMC9210726; doi:10.1186/s12875-022-01763-2)
Supplement: Supplementary file 1 — Additional file 1. [file 12875_2022_1763_MOESM1_ESM.pdf]

Supporting Information

1. Pharmaceutical Benefits Scheme (PBS) Medication Codes

Medication Class	Body System Code
Antihypertensives	C01BD C07A C08C C08D C09A C09B C09C C03A C03B C03D C03E C04A C02A C02C C02D C02K C02KX C09D
Statins	C10AA
Other lipid	C10AB C10AC C10AX
Statins + antihypertensives	C10BX
Statins + hypoglycaemic	A10BH
Statins + other lipid	C10BA

2. Boosted Regression Tree (BRT) Influence Plots

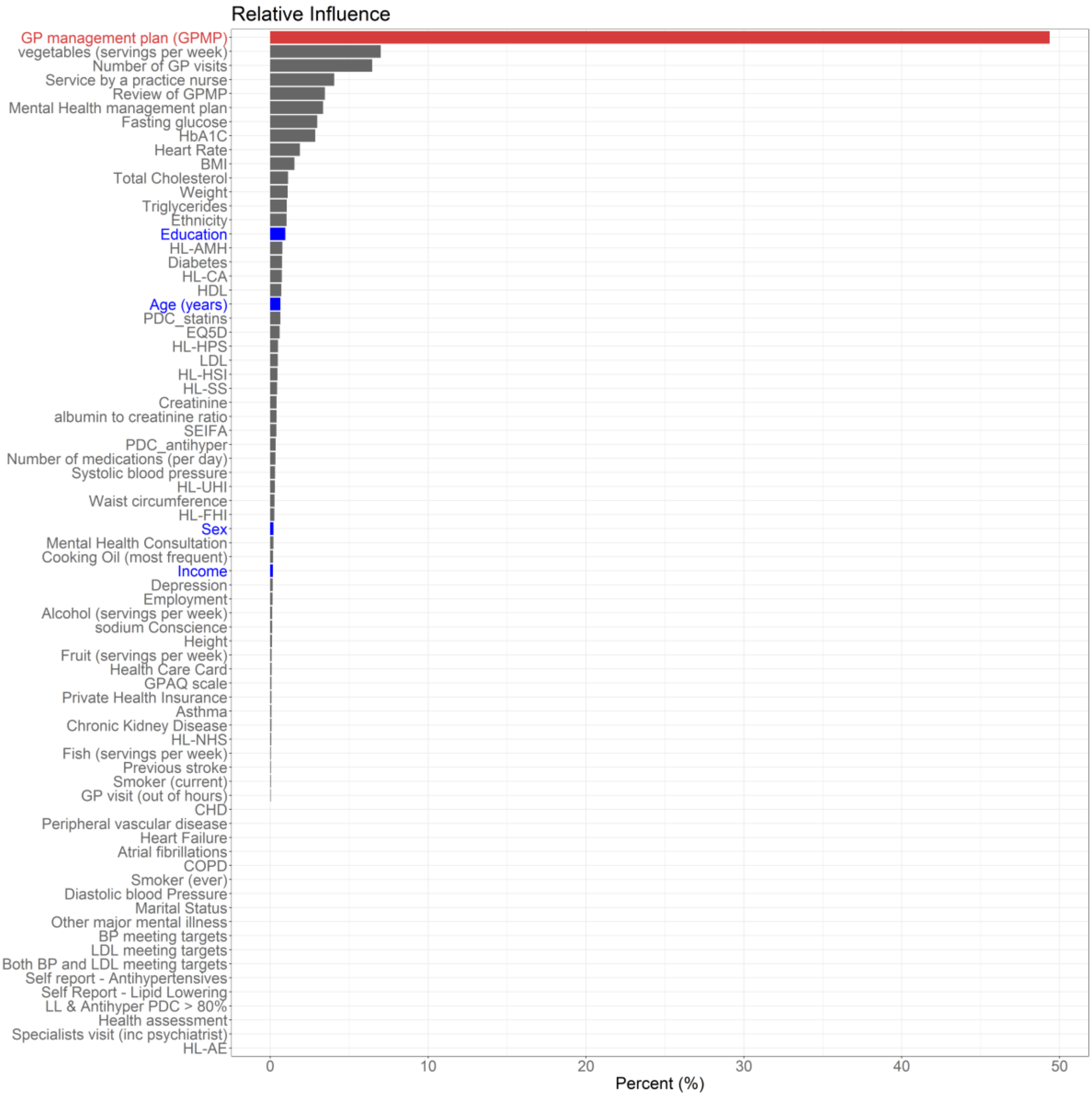

(a) Engagement with allied health services BRT influence plot.

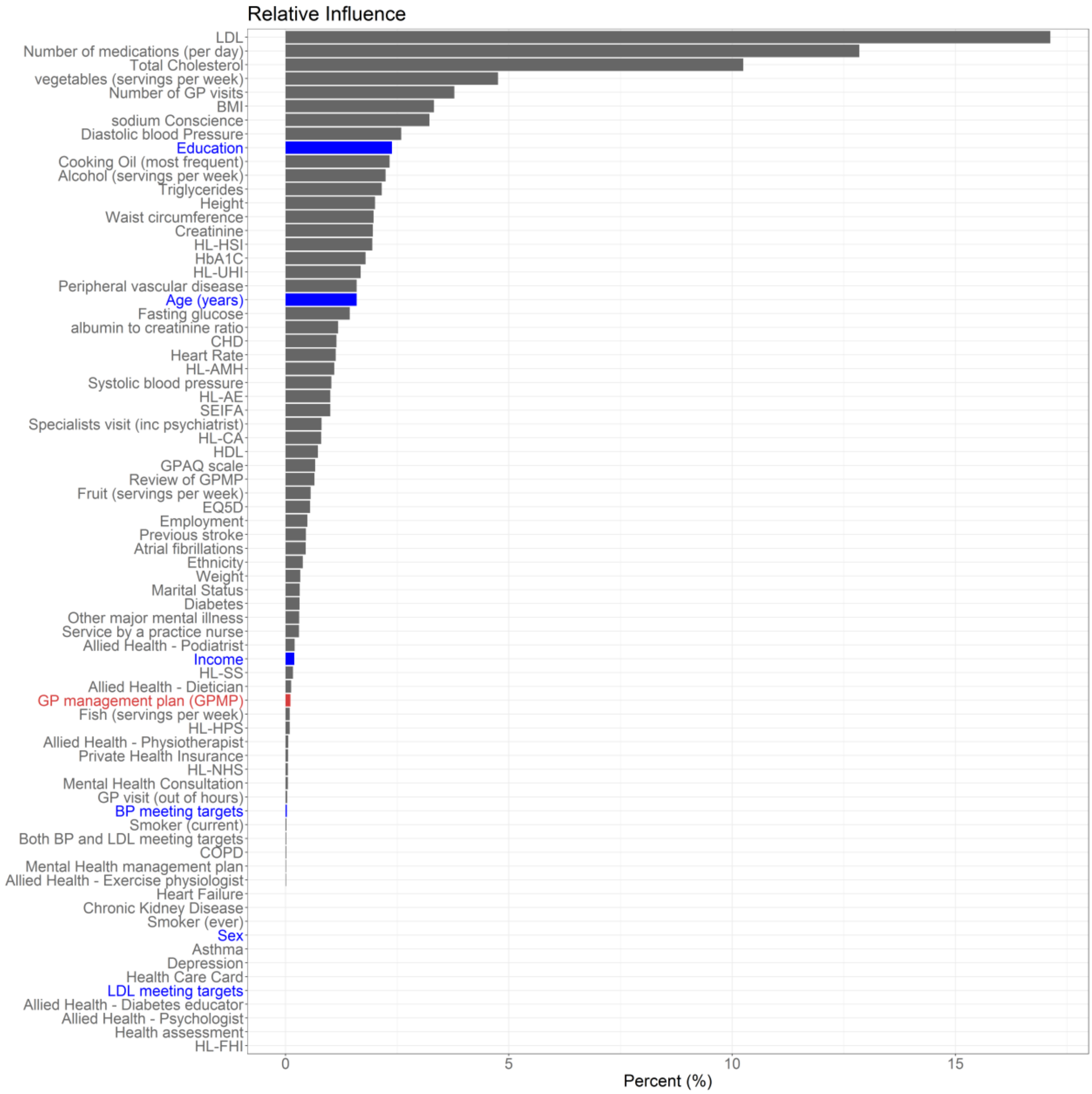

(b) Adherence to dual cardiovascular medications BRT influence plot.

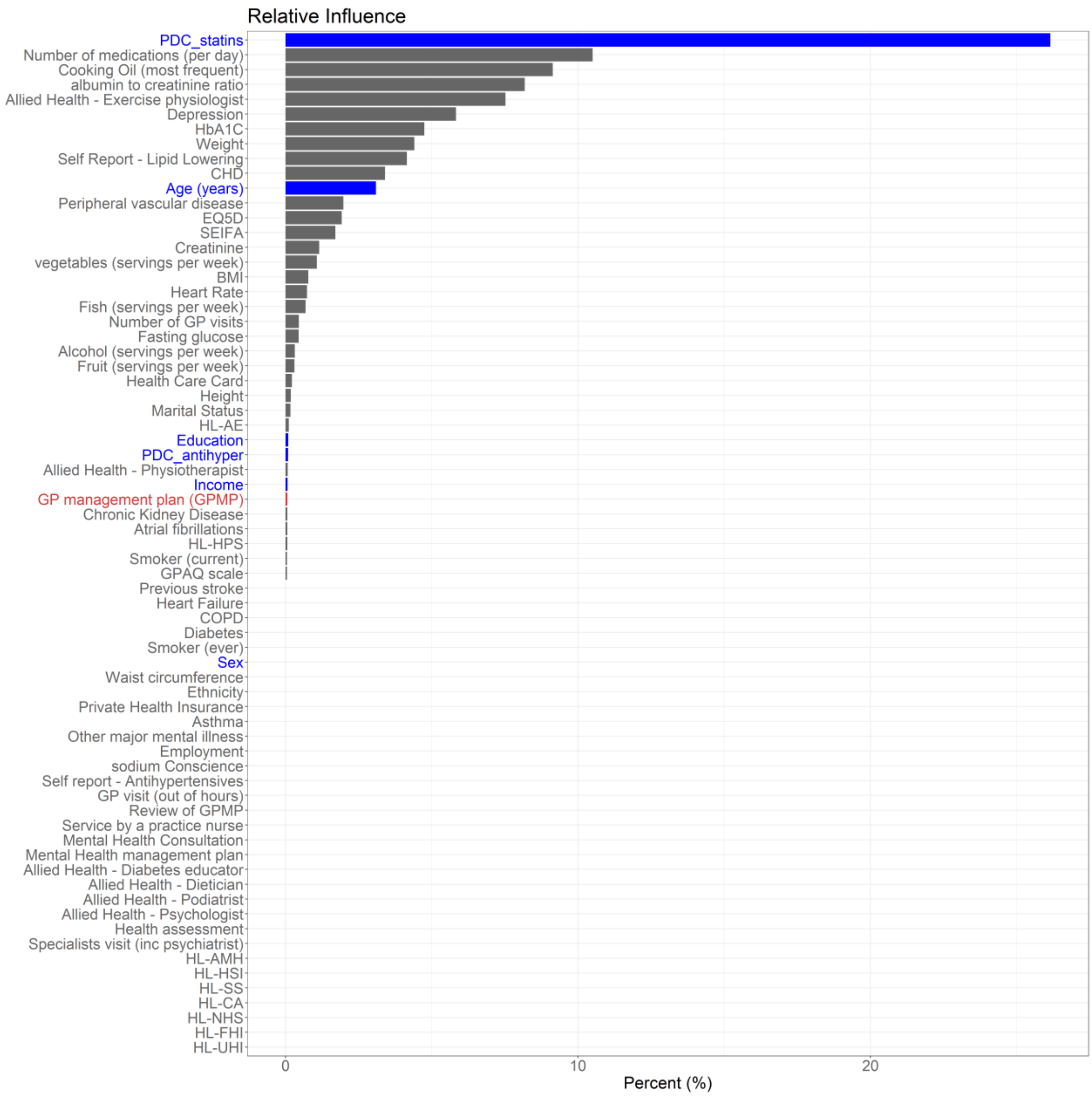

(c) Attainment of blood pressure and LDL cholesterol targets BRT influence plot.

3. Regression models using GPMP review

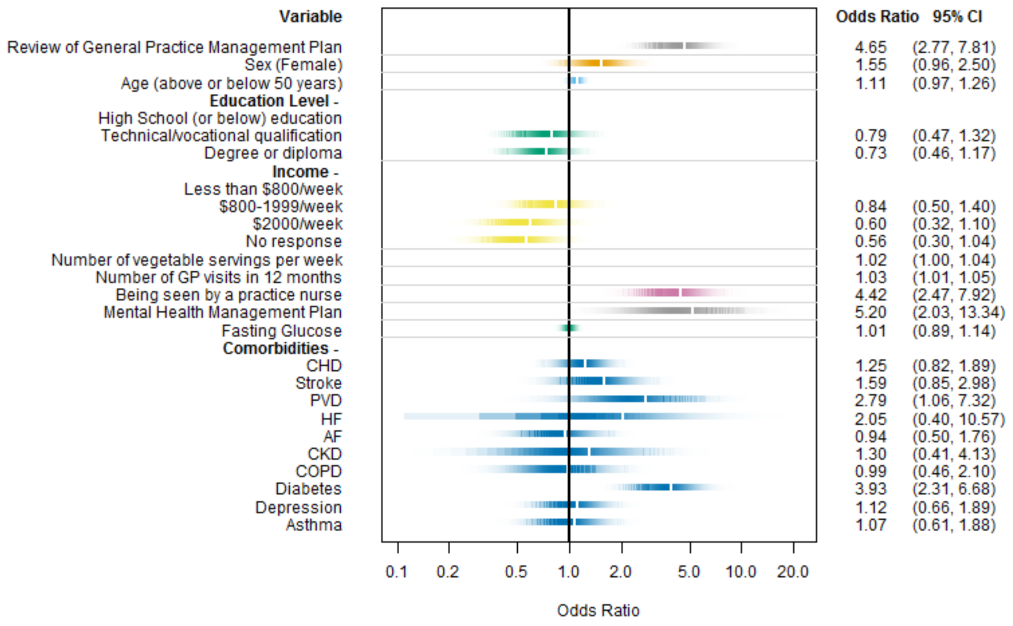

(a) Multivariable regression model for allied health service use using GPMP review.

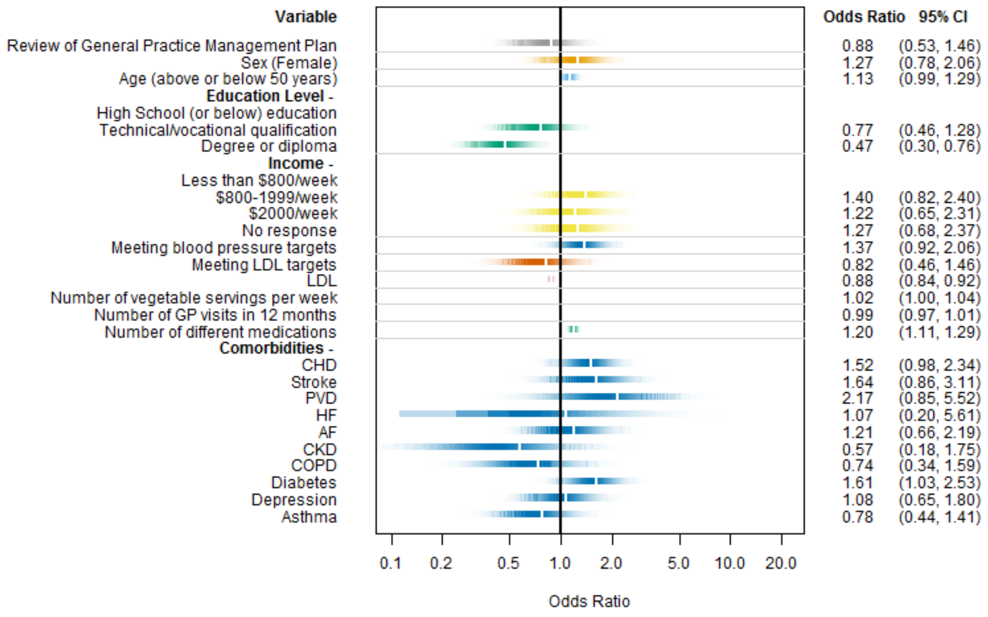

(b) Multivariable regression model for dual adherence to cardiovascular medications using GPMP review.

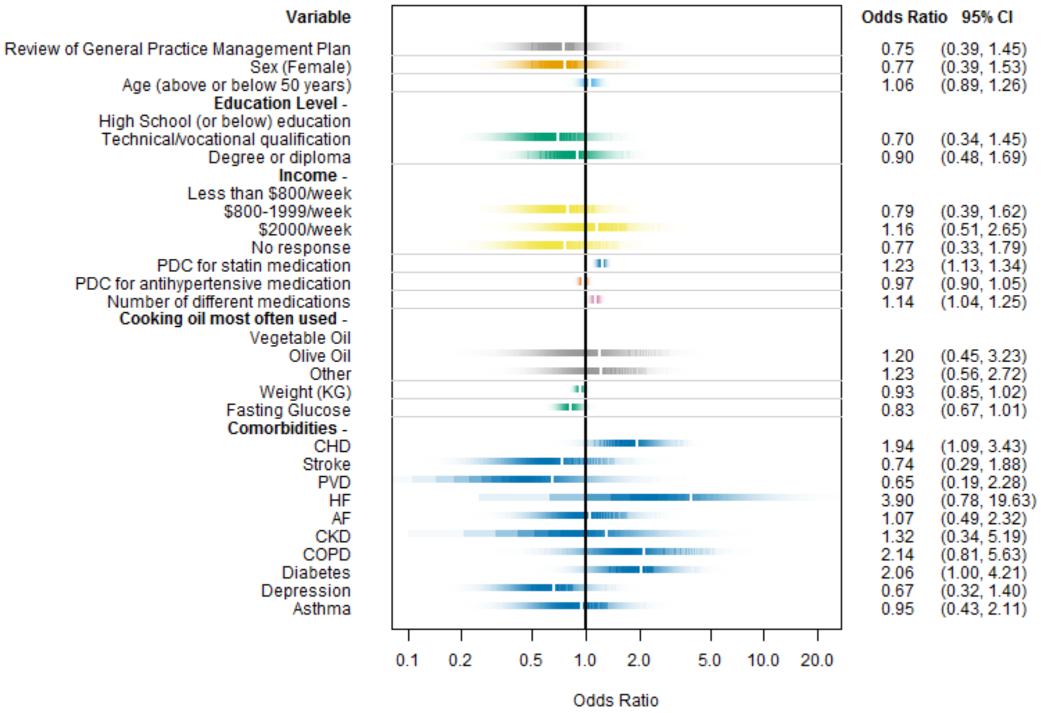

(c) Multivariable regression model for concurrent attainment of blood pressure and LDL cholesterol targets using GPMP review.
